# Supplementary material for: Demonstration of the Holonomically Controlled Non-Abelian Geometric Phase in a Three-Qubit System of a Nitrogen Vacancy Center
Source: Entropy (Basel). 2022 Nov 2;24(11):1593. doi: 10.3390/e24111593 (PMC9689909; doi:10.3390/e24111593)
Supplement: Supplementary file 1 [file entropy-24-01593-s001.zip › entropy-1874875-supplementary.pdf]

entropy

MDPI

Supplementary Information: Demonstration of the holonomically controlled non-Abelian geometric phase in a three-qubits system of an NV center

Shaman Bhattacharyya and Somnath Bhattacharyya*

Nano-Scale Transport Physics Laboratory, School of Physics, University of the Witwatersrand, Johannesburg, 1 Jan Smuts Avenue, Wits 2050, South Africa
* Correspondence: somnath.bhattacharyya@wits.ac.za

Methods: All the gates that are used to perform the holonomic control can be expressed as single-qubit U1, U2, and U3 gates. No multi-qubit gates have been used. This is because all the gates have to be consistent with the quantum Rabi model and also for a gate to be holonomic the gate must cause the qubit to rotate in any arbitrary axis while creating a geometric phase. Using a multi-qubit gate introduces the Dicke effect since the number of qubits is greater than 1. This is beyond the scope of our work. The fidelity of single-qubit gates is usually higher than a multi-qubit gate for these types of experiments.

Two qubits: After making the rotating approximation, the Hamiltonian acting on this system can be expressed in the rotating frame as:

$$H = \sum_{i=1}^{4} |\omega_i|\mathrm{i} >< \mathrm{i}| + \frac{i}{2}\left(\Omega_{P_1} e^{-iV_{P_1}t} - \Omega_{P_2} e^{-iV_{P_2}t}\right)(|1 >< 3| - |1 >< 4|)$$
$$-\frac{i}{2}\left(\Omega_{S_1} e^{-iV_{S_1}t} - \Omega_{S_2} e^{-iV_{S_2}t}\right)(|2 >< 3| + |2 >< 4|) \quad \text{(S1)}$$

The Hamiltonian in the interaction frame can be expressed as

$$H_I = \begin{pmatrix} 0 & 0 & i\Omega_{p1}/2 & i\Omega_{p2}/2 \\ 0 & 0 & -i\Omega_{s1}/2 & -i\Omega_{s2}/2 \\ -i\Omega_{p1}^*/2 & i\Omega_{s1}^*/2 & \Delta_1 & 0 \\ -i\Omega_{p2}^*/2 & i\Omega_{s2}^*/2 & 0 & -\Delta_2 \end{pmatrix} \quad \text{(S2)}$$

The norm of the return amplitudes for the 2 qubits (4 levels). NV center as functions of time as described by the four-level interaction Hamiltonian H for the states |00>, |01>, |10>, and |11>, respectively. It is important to note that the amplitude of states |10> and |11> are the same (Figure S1a, b). To perform the calculations, we assume that the system is initially at the state |1>.

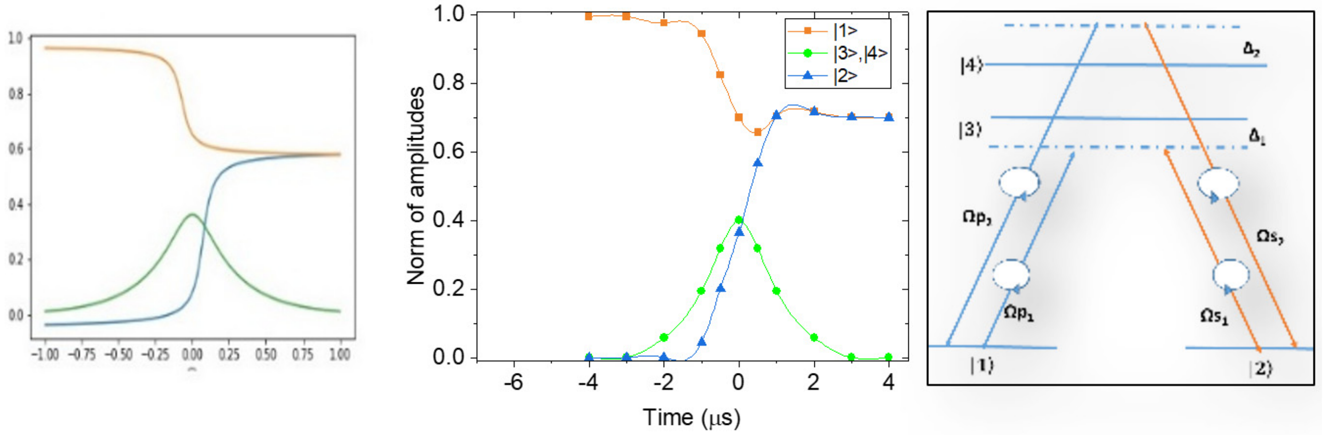

Figure S1. The norm of amplitudes for state vs. time shows the evolution of the states over time illustrating aπ/2 rotation of a four-level NV centre on a quantum computer. The blue line and triangles represent the state |2>. The orange line and squares represent the state |1>. The green line and

circles represent the states |3> and |4>. The states |1> and |2> are both degenerate. The Rabi frequencies of the NV center are both shown as functions of time which are already shown in Ref. [9]. Inset: The evolution of the states over time according to the model described by the interaction Hamiltonian given in Equations (S1) and (S2) and the energy level diagram. The results of [12] were reproduced.

Then, we solve the four-level interaction Hamiltonian using a time-dependent Schrödinger equation. We set ω = 20 MHz and α = 1.1 MHz. We then plot the norms of the amplitudes for all 4 states. It can be noted that the amplitudes for the |3> and |4> states follow the same trend and no populations of these two states exist both before and after the holonomic control pulses. For state |1> the amplitude decreases from 1 to about $1/\sqrt{2}$. The amplitude for state 01 increases to $1/\sqrt{2}$ from 0. This result is consistent as it shows that the qubit was rotated by $\pi/2$ about the x-axis. Figure S1 and inset show the evolution of the system over time to the exact equal superposition required after a $\pi/2$ rotation. The link between the four-level model and the states depicted here can be shown on two Bloch spheres, with $|1\rangle = |00>, |2\rangle = |01>, |3\rangle = |10>,$ and $|4\rangle = |11>$.

From the plotted return probabilities of each of the states in Figure 2b (in the main text) for the period over which the pulses are applied, it can be seen how the holonomic rotation of the one qubit is related to the rotation of the other qubit when the paths are concatenated. The return probabilities of the |3> and |4> states (shown by the orange line in Figure 2b) over this period start at zero, rise to a maximum at t = 0, and fall off to zero again, indicating a rotation about a full loop with a solid angle of $\pi/2$. This geometric phase corresponds to the $\pi/2$ R_x rotation of the first qubit, as seen by the return probabilities of the |1> and |2> states – the P_r of the |1> state is initially at 1, but decreases to $1/\sqrt{2}$, while that of the |2> state is increased from 0 to $1/\sqrt{2}$ after the pulses have been applied. This agrees with the result from Lu et al. who demonstrated such a two-qubit holonomic control using a 4 level NV center system [12]. The simulated reproduction of these results resembles those of Lu et al. more closely than the emulated reproduction of the results, as the quantum emulator used does not have NV center qubits, but rather uses superconducting qubits [12]. This, along with other sources of error such as noise, is why the discrepancy in the emulated results is higher than in the simulated results. This discrepancy is further shown in Figure S1 and inset, where the population of the |00> state is plotted with time after the concatenation of the rotation paths. In the absence of noise, there is very little fluctuation in the P_r of the |1> state (the orange line), but as soon as noise is introduced in the emulation, the population of the |1> state oscillates due to the geometric phase with a magnitude that depends on the level of degeneracy. The comparison to the case with no noise is important as it allows the phase to be indirectly measured.

Two qubit holonomic gates: Abelian phases arise from non-degenerate systems due to the adiabatic evolution of the phase Hamiltonian. The exact opposite is true for degenerate states. An experiment was conducted, and it was discovered that the nature of such geometric phases is non-Abelian. As mentioned in the introduction NV centers are an ideal choice for holonomic control due to their coherence times at room temperatures. Another key advantage of NV centers is the fact that the sublevels of the NV center can be manipulated via an external magnetic field that allows for the variation in degeneracy between eigenstates. Now degeneracy is possible for SU(2) and higher-order systems only. In this simulation, the axis of the first qubit is located in the nitrogen atom while the axis of the second qubit extends from the nitrogen atom to the adjacent vacancy. We use the lab frame reference for the qubit states. Without an external magnetic field, the ground states ±1 states are degenerate. A magnetic field applied to the z-axis of the second qubit induces Zeeman splitting. If no magnetic field is applied the resulting phase is Abelian since the state m_s = 0 is non-degenerate. To demonstrate the phase creation in this work we start by letting the ground states of the first qubit (|1> and |2>, respectively) to be degenerate. Now in an experiment degeneracy is maintained by rotating the NV center and the magnet together. In IBM QE we use two qubits to represent the four levels of the

NV center. As with the single-qubit scenario, a unitary operator U is implemented keeping one of the rotation angles θ or φ constant. The operator U is expressed in terms of R_X, R_Y, and R_Z gates in IBM QE because these operations are unitary and are expressed in terms of both θ and φ. We then create two different rotation paths.

We implement each path on each qubit. The starting point of the paths is created by applying an R_X (π/3) rotation which is implemented by setting θ to -π/2 and φ to π/3 on both qubits. The first path is a complete azimuthal rotation around the Bloch sphere. The phase matrix is approximated as: U= (−0.609+0.321i0.786+0.1i−0786+ 0.1i−0.609+0.321i). The second path is a complete loop defined by θ and φ. The closed loop is created by a complete rotation around the y-axis of the second qubit. The coordinates of this rotation given in terms of φ and θ are given by (θ, φ) = [(π/3, 0), (0, π/3), (-π/3, 0), (0, -π/3)]. Now both paths accumulate a geometric phase. Since the excited states are non-degenerate and only dφ or dθ is nonzero the resulting geometric phase is Abelian. We also interchange the paths to observe any difference in the geometric phase. Now a disadvantage of SU(2) and higher-order systems is that the resulting geometric phase cannot be directly observed. In IBM QE we attempt to show this geometric phase by measuring the qubits in the |z> basis to obtain the density of the ground states. We also determine the expected density of states in the absence of the geometric phase and decoherence.
